# The relationship between spatiotemporal gait parameters and cognitive function in healthy adults: protocol for a cross-sectional study

**DOI:** 10.1186/s40814-022-01122-z

**Published:** 2022-07-25

**Authors:** Tatsuya Fukuoka, Shun Irie, Yoshiteru Watanabe, Toshiki Kutsuna, Akiko Abe

**Affiliations:** 1 R&D division, Xenoma Inc, Tokyo, 143-0013 Japan; 2 Division of Smart Healthcare Research, Dokkyo Medical University, 880 Kita-Kobayashi, Mibu, Tochigi, 321-0293 Japan; 3 Major of Physical Therapy, Department of Rehabilitation, School of Health Sciences, Tokyo University of Technology, Tokyo, 144-8535 Japan; 4 Major of Occupational Therapy, Department of Rehabilitation, School of Health Sciences, Tokyo University of Technology, Tokyo, 144-8535 Japan

**Keywords:** Mild cognitive impairment, Motoric-cognitive risk syndrome, Locomotor functions

## Abstract

**Background:**

Motor dysfunctions, such as slower walking speed, precede the occurrence of dementia and mild cognitive impairment, suggesting that walking parameters are effective biomarkers for detecting early sub-clinical cognitive risk. It is often also concurrent with self-complained cognitive dysfunction, called motoric cognitive risk (MCR) syndrome. Our preliminary study found several walking parameters, obtained by a three-dimensional motion capture system, to be correlated with computer-based assessments of various cognitive function modalities, although the sample size was small. The Cognitive-Gait (CoGait) Database Project, described in the current protocol, aims to establish a database of multi-dimensional walking and cognitive performance data, collected from a large sample of healthy participants, crucial for detecting early sub-clinical cognitive risk.

**Methods:**

We will recruit healthy volunteers, 20 years or older, without any neurological musculoskeletal or psychiatric disorders. The estimated sample size is 450 participants, including a 10% attrition rate. Using computer-based cognitive assessments, participants will perform six tasks: (i) the simple reaction time task, (ii) Go/No-Go task, (iii) Stroop Color–Word Test, (iv) N-back test, (v) Trail Making Test, and (vi) digit span test. We will also conduct paper-based cognitive assessments such as the Mini-Mental State Examination, Montreal Cognitive Assessment, and the Geriatric Depression Scale-15 for assessing MCR. Gait will be measured through joint kinematics and global positioning in participants’ lower legs while walking at a comfortable and faster pace, using pants with an inertial measurement unit-based three-dimensional motion capture system. Finally, we will establish a prediction model for various cognitive performance modalities based on walking performance.

**Discussion:**

This will be the first study to reveal the relationship between walking and cognitive performance using multi-dimensional data collected from a large sample of healthy adults, from the general population. Despite certain methodological limitations such as the accuracy of measurements, the CoGait database is expected to be the standard value for both walking and cognitive functions, supporting the evaluation of psychomotor function in early sub-clinical cognitive risk identification, including motoric-cognitive risk syndrome.

**Supplementary Information:**

The online version contains supplementary material available at 10.1186/s40814-022-01122-z.

## Background

Motoric-cognitive risk (MCR) syndrome, characterized by self-reported cognitive complaints and slower walking speed, is associated with an increased risk for dementia and mild cognitive impairment (MCI) [1–4]. Detection of MCR syndrome can facilitate early intervention, such as pharmacological treatment, rehabilitation for sustained cognitive and physical functions, and prevention of MCI and dementia [5–9]. In addition to patient education for lifestyle modification, addressing issues such as diet, physical, and social activities reduces the risk of these cognitive disorders [10–15]. In a recent survey, the incidence rate of MCR in people in their 60s was 54.9 per 1000 persons, which strongly suggests that preventive activities for these cognitive disorders should be started at working age, before the initial presentation of early cognitive decline [16].

There are many risk factors for dementia, such as genetic factors, lifestyle habits, sleep quality, education, and physical and social activities, even in the absence of detectable cognitive risk [6, 7, 10, 11, 17–19]. Thus, we argue that cognitive risk screening in healthy participants requires additional multimodal parameters. Establishing a database of multimodal parameters that include these risk factors is necessary to distinguish individuals with higher cognitive risks from the general population.

**Fig. 1:**
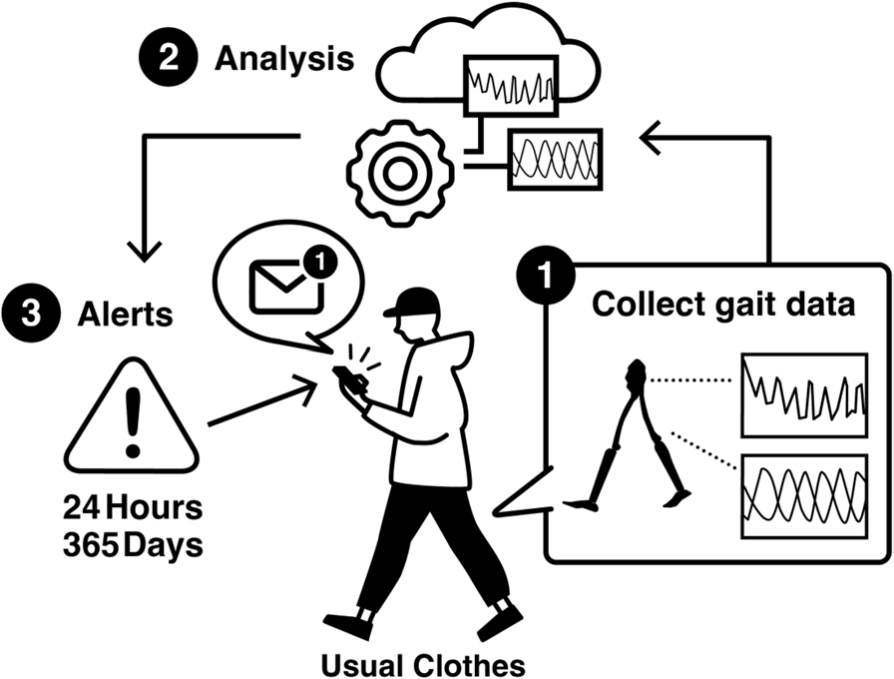
Schematic diagram for the utilization of the database of cognitive and walking functions. This is the ultimate goal of this study: First, a person wears the IoT clothes that can collect the walking parameters in daily environments (1). Parameters of walking functions are uploaded to the cloud, and cloud computing is performed to define the risks of cognitive impairment and frailty (2). When the risks are above standard value, notifications will be sent to the person’s smartphone (3). Finally, that person will start preventive activities according to the notifications

Specifically, walking performance is the most notable marker of cognitive risk [20, 21]. Although walking speed is a known risk factor for cognitive decline, what domains of walking parameters (i.e., cadence, speed, toe clearance, joint angle) relate to early cognitive decline remain unknown. Therefore, a motion capture technique that can collect information on various parameters of walking functions is a potential screening methodology for early cognitive decline. Motion capture systems are divided into two categories: one is a conventional optical system with infrared cameras and reflective markers, and the other is an IMU (inertial measurement unit)-based system. It is easier to utilize the IMU-based motion capture system to monitor walking functions in daily environments because it is easy to assemble and measure in any place compared to the conventional optical motion capture system [22] (summarized in Table 1). Ultimately, we aim to establish a database of cognitive and walking functions, enabling us to detect cognitive risks in earlier stages based on walking performances in daily environments collected by the wearable IMU-based motion capture system, including the e-skin MEVA (Fig. 1). The conventional optical motion capture system cannot be utilized to achieve our aim as it requires special facilities and techniques. Even if there are limitations related to the accuracy of tracking data when using the IMU-based system, this system is best suited for our study objectives.

**Table 1:** Comparison of specifications between conventional optical and IMU-based motion capture system

	Optical (conventional) system	IMU-based system
Accuracy	High	Low
Cost	High	Low
Place	Specialized facilities	Anywhere
Time	Long	Quick
Daily use	No	Yes (as wearable devices)

For cognitive assessments, MMSE (Mini-Mental State Examination) and MoCA-J (Japanese version of Montreal Cognitive Assessment) are frequently used to detect dementia and MCI. Although these are powerful screening tools for the screening of dementia and MCI, ceiling effects have frequently been reported among healthy participants in these paper-based tests [23–26]. Thus, web-based cognitive assessment has recently gained attention, as it is more feasible for detecting earlier stages of cognitive decline as compared to paper-based testing [27].

Therefore, we preliminarily examined walking parameters using the e-skin MEVA and cognitive function scores, using web-based assessments (CogEvo, Total Brain Care, Kobe, Japan) in healthy participants (5 women and 6 men; 28–80 years). The CogEvo consisted of five cognitive function domains: spatial recognition, orientation, memory, planning, and attention. These domains were measured by pattern recognition task, confirming the date, flashlight task, maze task, and Trail Making Test, respectively [28, 29]. Resultantly, our preliminary observations, even in a small sample, showed a significant relationship between some cognitive and walking performance modalities in healthy volunteers (see Supplementary Figure S1, Additional file 1). From these results, we establish the study protocol to more comprehensively investigate the relationship between multi-dimensional walking parameters and cognitive function using a custom-based web-application which comprises more cognitive domains than CogEvo (see “Methods” section) and which can be utilized to predict and screen for cognitive function decline. Our study will collect the detailed walking parameters and cognitive assessment scores of a sample of healthy volunteers, aged 20 years or older, to establish more accurate predictive models for cognitive function.

## Methods

### Study design

The current study, called the Cognitive-Gait Database (CoGait) project, will follow a cross-sectional design aimed at elucidating the relationship between walking and cognitive performance in healthy adults. The sample size was estimated to be 410 persons, calculated using the effect size (*f* = 0.074) of a similar study [20] under the following conditions: multiple linear model, *df* = 24, *P* = 0.01, 1-β= 0.8. The upwardly corrected sample size of 450 people accounts for an attrition rate of 10%. The sample size calculation was performed using G*power [30, 31]. Before submitting this article, we confirmed whether the design was suitable for the STROBE checklist for cross-sectional studies (see Additional file 2). After publication of our study results, we will immediately publish all datasets except personal information in a secured database server.

### Study setting and recruitment

Healthy volunteers, older than 20 years and without any neurological or musculoskeletal disorders, will be recruited for the study. The research team consists of a research scientist at Xenoma Inc. (TF), as well as faculty members at the Tokyo University of Technology (YW, TK, and AA), and Dokkyo Medical University (SI). To recruit participants, we will post advertisements about the study through our partners, such as universities (Tokyo University of Technology and Dokkyo Medical University), local governments (Ota-City in Tokyo), local corporate associations, and companies for recruitment testing. To reduce any age-related bias, our advertisement partners will be assigned to collect data from different age generations. For example, younger participants from universities, younger to middle-aged individuals from the local corporate associations, and the elderly from the local governments. Our advertisements will assure prospective participants of their right to withdraw at any time and the financial benefit for participating (approximately 1000 JPY). The study interval is set to 3 years. If we are unable to obtain 450 samples within this time frame, the study will be concluded, and the database will be published as soon as possible. The location of measurement will be gymnasiums and halls in universities and local governments.

### Study participants

#### Inclusion criteria

Healthy participants older than 20 years, without any neurological, musculoskeletal, or psychiatric disorders potentially affecting walking and cognitive functions, will be eligible for participation. Only native Japanese speakers will be recruited. Informed consent will be sought from all participants before they declare their medical histories. In the absence of exclusion criteria (see the “Exclusion criteria” section), participants’ walking, and cognitive performance will be measured (see the “Measures” section).

#### Exclusion criteria

Participants will be excluded from the study if their medical histories include the disorders or conditions listed in Table 2. Participants with visible abnormalities in walking function (i.e., a mobility function score of <7 on the Functional Independent Measure), as assessed by a skilled physician, physiotherapist, nurse, or research scientist, will also be excluded [32]. Additionally, participants with cognitive problems (i.e., MMSE <24 and MoCA-J ≤25) or self-complaint cognitive dysfunctions assessed by the Geriatric Depression Scale 15 (GDS-15) will be excluded [33].

**Table 2:** Exclusion criteria

Exclusion criteria	
People who cannot walk independently	
People with any amputations.	
People with disabilities in vision, hearing, and/or equilibrium.	
People who cannot use an electronic tablet device owing to disabilities in their upper limbs.	
People at a high risk of falling.	
People with detectable (MMSE <24 and MoCA-J ≤25) or self-complaint cognitive dysfunctions assessed by GDS-15.	
People with other orthopedic, neurological, or psychiatric disorders that potentially affect walking and cognitive functions (i.e., osteoarthritis, stroke, depression).	

### Measures

#### General procedure

**Fig. 2:**
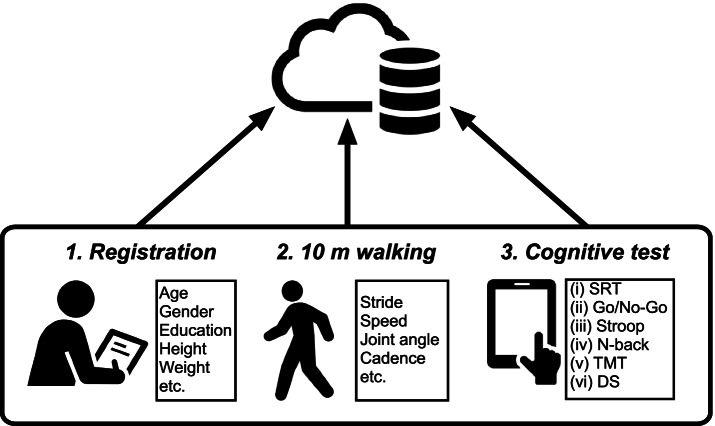
Schematic diagram of the experimental procedure. The experiments consist of four phases or sessions: informed consent, registration of personal information (i.e., height, gender, and date of birth), gait measurement with e-skin MEVA, and cognitive assessment using the tablet. Datasets will be automatically uploaded to online cloud storage

First, we will obtain written informed consent from all participants. Second, we will obtain their personal information (Table 3), medical histories using the checklists, MMSE and MoCA-J scores, and the questionnaire for subjective memory complaints using GDS-15, in order to exclude participants with pre-diagnostic cognitive disorders (Table 2) [33]. Medical history includes neurological, orthopedic, and psychiatry disorders that potentially affect the walking and cognitive functions (see the “Exclusion criteria” section) [34, 35]. Medical histories will be recorded, and cognitive assessments will be performed by a skilled physician, physiotherapist, nurse, or research scientist face-to-face. Unique personal identities (IDs) will be generated for participants who do not meet the exclusion criteria and printed as QR codes, required during registration for the walking and cognitive assessments. Upon receiving their IDs, participants’ walking will be measured (see the “Gait measurement” section). After a 10-min break, they will participate in the cognitive assessment (see the “Cognitive assessment” section). The walking and cognitive function datasets will be securely stored in online cloud storage. An overview of the study procedures and measurements is presented in Fig. 2.

**Table 3:** Items for collecting participants’ personal information

Personal information	Body information
Name	Body height
Gender	Body weight
Date of birth	Sex
Contact	Other disorders
Education	Medications
Mother tongue

#### Gait measurement

**Fig. 3:**
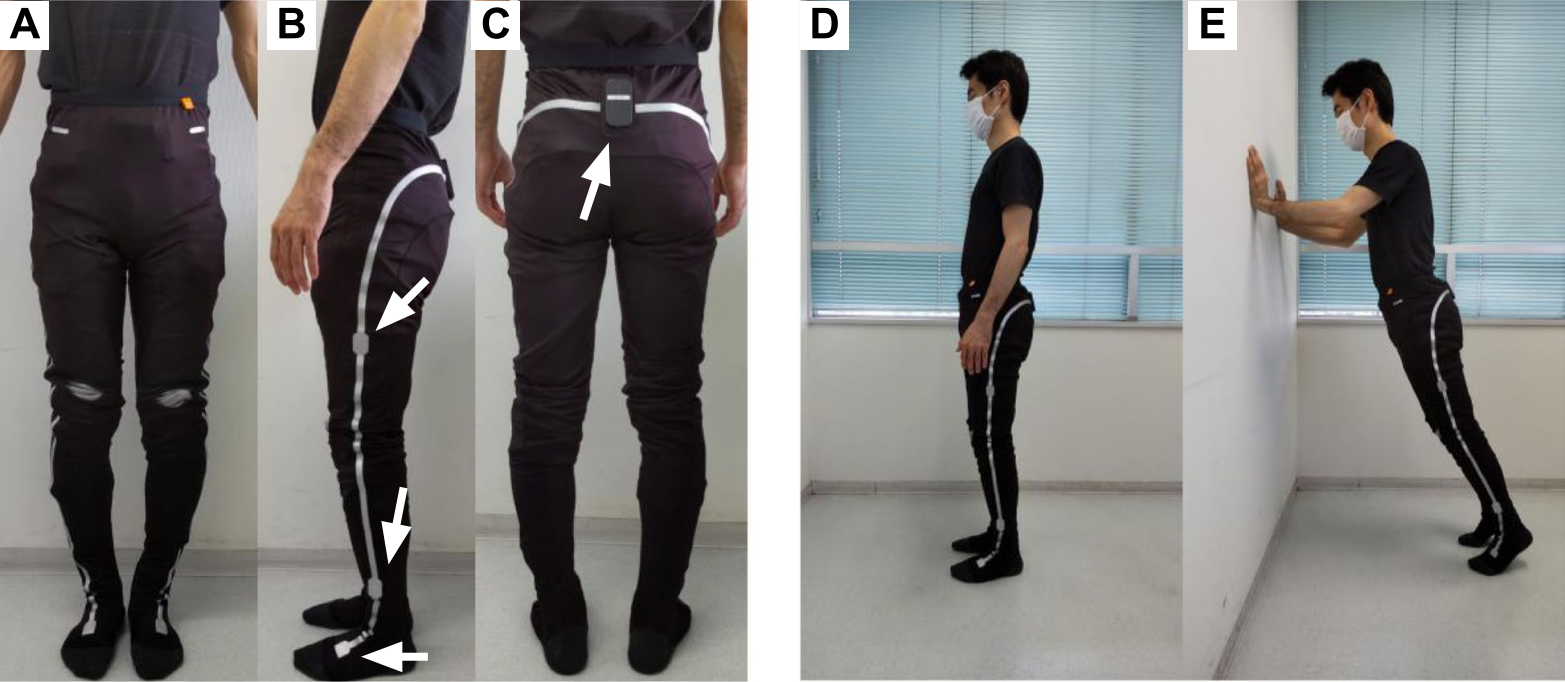
The wearable motion capture system “e-skin MEVA.” **A**–**C** The IMU pants, from the frontal (**A**), lateral (**B**), and posterior (**C**) views. IMU sensors are placed on the lateral surfaces of the upper and lower legs, and the dorsal surfaces of the foot and sacrum. The sensor on the sacrum is equipped with a removable attachment, with Bluetooth® wireless communication. **D**, **E** The poses for calibration. Prior to the measurement, participants must pose in two ways: leaning forward (**D**) and standing upright (**E**)

We will measure walking parameters using pants, fitted with seven inertial measurement unit (IMU) sensors (e-skin MEVA; Xenoma Inc., Tokyo, Japan), located in the e-textile segments, as shown in Fig. 3A–C. IMU sensors contain triaxial accelerometers and triaxial gyroscopes, enabling the estimation of three-dimensional joint kinematics and global positioning according to a known algorithm [36]. Prior to the development of the current study protocol, we conducted a validity check comparing joint angles across the e-skin MEVA and the conventional optical motion capture system (VICON Nexus ver. 2.1.1, VICON, Oxford, UK). Overall, the systems were comparable (root mean square error: 3.57±1.50°; *r* = 0.96±0.03, on both sides of the hip, knee, and ankle joints; see Supplementary Figure S2, Additional file 1).

To calibrate the three-dimensional model calculation prior to gait measurement, each participant will be asked to adopt two postures: leaning forward with their hands pressed against a wall (Fig. 3D) and standing upright (Fig. 3E). Next, the participants will be asked to walk in a straight line on a 16-m walkway, including 3-m inlet zones, at the start and end points. The measurements will be conducted under two conditions: *fast* (maximum speed) and *comfortable* (self-selected speed). Participants will practice the walking task under each condition several times before the measurements to ensure they understand the requirements of the experimental tasks. In the *fast* condition, we will instruct participants to walk at their maximum speed, without running or falling. In the *comfortable* condition, we will instruct them to walk at their regular, comfortable speed.

The measurement datasets will comprise raw IMU sensor signals (acceleration and angular velocity), global positioning of each sensor and anatomical landmark, and joint angles in the pelvis, hip, knee, and ankle (347 parameters in total). The datasets will be automatically uploaded to cloud storage (Fig. 2). The data processing methods are described below (see the “Data analysis” section).

#### Cognitive assessment

Cognitive assessments will be conducted using the conventional paper-based screening tests: MMSE, MoCA-J, and GDS-15, and a custom-developed web-based software application. Computer-based cognitive assessments cover a wide range of cognitive functions and minimizes floor and ceiling effects [37]. Moreover, such assessments can collect data not only in terms of accuracy of each task, but also in terms of temporal, spatial, and spatiotemporal domains, differentiating them from conventional paper-pencil-based cognitive assessments [38, 39]. Thus, we believe that the combination of conventional paper-based and web-based assessments will assist further studies extended to MCI and MCR.

The software was coded using JavaScript® and runs on a web browser (Safari, Apple, Cupertino, CA). To ensure visual conformity, all tests will be conducted using tablets with the same model number (iPad (8th), Apple, Cupertino, CA). The measure consists of six subtests: (i) simple reaction time (SRT) task, (ii) Go/No-Go task, (iii) Stroop Color–Word Test, (iv) N-back test, (v) Trail Making Test (TMT), and (vi) digit span (DS) test. During the tests, the tablets will be positioned in a landscape orientation and tilted at 20°. Participants’ right index fingers will be placed 2.0 cm behind the tablet. Before any tests, all participants will practice the tasks at least twice, with verbal instructions from the expert staff, using a tablet.

##### (i) Simple reaction time task

**Fig. 4:**
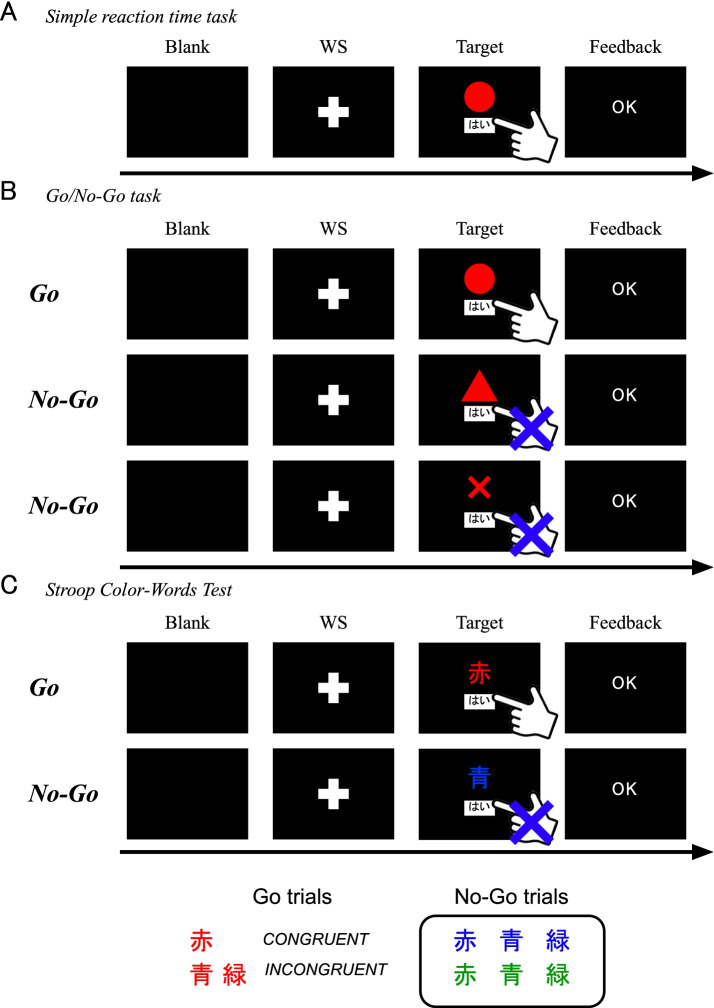
Cognitive assessments using the reaction time paradigms. **A ***Simple reaction time (SRT) task*: In addition to the presence of the fixation point, the warning signal (WS) rings (1000 Hz, 50 ms). Participants should tap “はい (Yes)” as soon as possible after the WS. Feedback is presented after the responses. **B ***Go/No-Go task*: The flow of the task is almost the same as that of the SRT. However, in this task, participants are asked not to respond to a non-target presentation (triangle and cross). **C ***Stroop Color–Word Test*: All experimental paradigms and flows are similar to those in the *Go/No-Go task*. Participants responded only to the red Chinese letters

The flow of the SRT task is illustrated in Fig. 4A. Participants will be asked to fix their gaze on the center of the white cross (fixation point), and after the warning signal (1000 Hz, 50 ms), the target signal (red circle) will appear on a black background at random timings (1–3 s after the warning signal). Participants will be asked to press the “はい (Yes)” button with their right index finger when the signal appears [40]. The SRT task comprises 10 trials.

##### (ii) Go/No-Go task

The flow of the Go/No-Go task is shown in Fig. 4B. The procedure of this task is similar to the SRT task, but the target signal is either a red circle, red triangle, or red cross, on a black background with a 2-s presentation time. The participants will be asked to respond only when the red circle appears on the display. The task consists of 10 trials each, for the “Go” and “No-Go” paradigms [41]. The Go/No-Go ratio was determined such that the reaction time was prolonged compared to SRT in healthy volunteers (*n* = 4; *M =* 38.5 years, *SD =* 12.2; Supplementary Figure S3A, Additional file 1).

##### (iii) Stroop Color–Word Test

The Stroop Color–Word Test was translated from a previous study [42] and converted into a digitalized test, using a tablet. The color words are displayed as target signals after the warning signal. The target signals are the Japanese words (*kanji*) [***RED***], [***BLUE***], and [***GREEN***], with the font color set to one of these colors, on a black background, with a 2-s presentation time (Fig. 4C). Participants will be asked to respond only when any word in a red font appears (Go trial). The Go trial consists of congruent (color and word match) and incongruent (color and word mismatch) conditions [43]. The ratio between congruent and incongruent groups is 1:1 [43], and the task consists of 10 trials each, for the Go and No-Go paradigms. In addition to the Go/No-Go task, the reaction time was prolonged compared to SRT in healthy volunteers (*n* = 4; *M =* 38.5 years, *SD =*12.2; Supplementary Figure S3A, Additional file 1).

##### (iv) N-back task

**Fig. 5:**
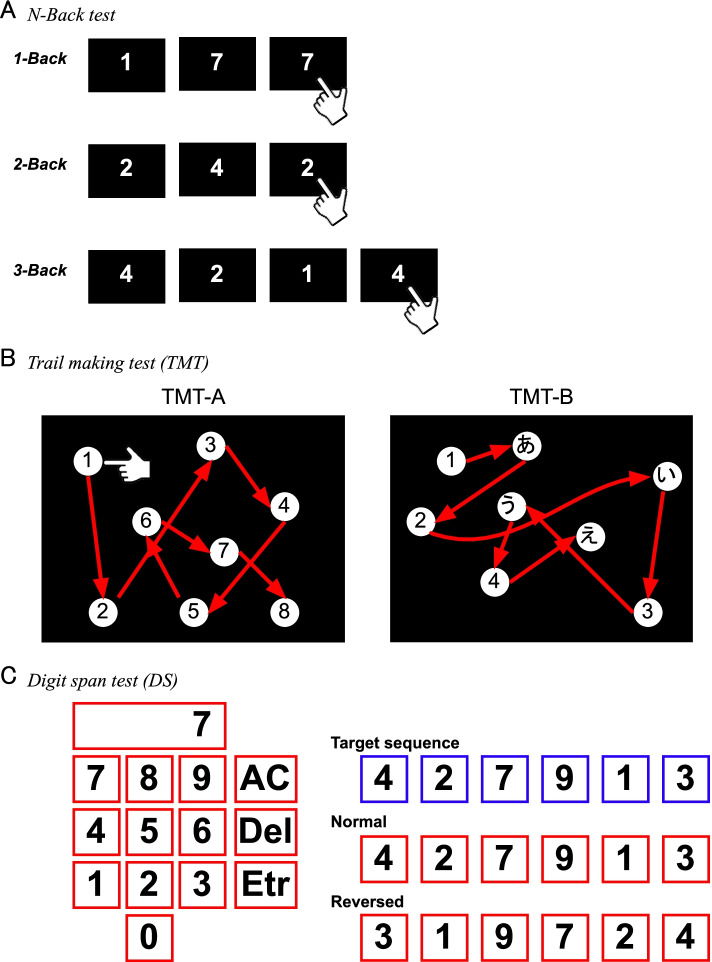
Cognitive assessments using the process time paradigms. **A ***N-back test*: The *N-back test* is one of the most established assessments of working memory capacity. Participants are asked to respond when the target signal is the same as the *last n* (1–3) digit number. **B ***Trail Making Test (TMT)*: The TMT consists of TMT-A (numeric numbers alone) and TMT-B (a mixture of numeric numbers and Japanese *kana* letters). In the TMT-A, participants are instructed to tap the numerical digits in ascending order. In the TMT-B, participants also tap the targets in ascending order, but they should tap the numerical digits and *kana* letters alternately. **C ***Digit span (DS) test:* This test uses a numeric keypad and numeric number indicator. Participants are asked to remember the target sequence of numerical digits individually shown in the indicator. After the presentation, they are asked to recall the sequence in the same or reversed orientation, using the keypad. After inputting the digit sequences, they tap the Enter (Etr) key. In addition, they can fix the input using the All Clear (AC) and Delete (Del) keys

The N-back task is a major approach used for assessing working memory capacity [44, 45]. Single-digit numbers will be displayed on the tablet as target signals (Fig. 5A), with a presentation time of 2 s for each target signal.

In the one-back condition, the participants will be asked to respond only when the target signal is the same as the last number displayed (congruent condition). In the two- and three-back conditions, they should respond only when the target signals are the same as the second-to-last and third-to-last numbers displayed, respectively. The frequency of the congruent condition will be set to 44% of the target signals, and the task consists of 15 trials for each n-back condition. In this condition, reaction times in healthy volunteers (*n* = 5; *M =* 34.4 years, *SD =* 4.98) were prolonged in the *N* = 1 and 2, compared to *N* =1 (Supplementary Figures S3B and C, Additional file 1).

##### (v) Trail making test

We adapted the Japanese version of the paper-based TMT, so that the data could be uploaded to our cloud storage [46]. The TMT consists of the TMT-A and TMT-B. The TMT-A contains 25 circled numbers, ranging from 1 to 25; participants will be asked to tap the circled numbers in order, from 1 to 25 (Fig. 5B). The TMT-B contains 13 circled numbers, ranging from 1 to 13, and 12 circled Japanese kana letters; participants will be asked to tap the numbers and letters following the rule 1-[あ]-2-[い]-3-[う]…[し]-13, corresponding to the original alphabet version of TMT-B (Fig. 5B) [46, 47]. In our adapted TMT, the participants will not need to draw a line on the screen to prevent misrecognition of tapping on non-target symbols. From preliminary experiments, the process time for the TMT-B in healthy volunteers (*n* = 4; *M =* 30.3 years, *SD* = 4.03) was markedly prolonged compared to that for the TMT-A, which was similar to previous results (Supplementary Figure S3D, Additional file 1) [48].

##### (vi) Digit span test

The DS test is a well-established measure of working memory capacity [49, 50]. The digit indicator and numeric keypad used in the DS are shown in Fig. 5C. Numerical digits are presented individually and sequentially, and the participants will be asked to remember the sequence of the presentation. Next, the participants will be asked to recall the sequence, using the numeric keypad. The DS test will be conducted under forward and backward conditions. The participants will be required to recall the sequences in forward or backward direction, depending on the task condition; sequence length ranges, from two to nine numerical digits, in both tasks. Participants will be required to perform the test under both conditions, and the trials will be repeated three times for each sequence length. When participants record three mistakes in the same sequence, the DS test will be completed. In our preliminary experiment with healthy volunteers (*n* = 4; 29.8±2.68 years), the matching rate for the backward condition was relatively low for longer target number sequences (Supplementary Figure S3E, Additional file 1).

### Data analysis

#### Gait analysis

From the gait measurement datasets, we will calculate the general walking parameters, such as stride length and minimum toe clearance, using the built-in software (e-skin LETS WALK, Xenoma Inc., Tokyo, Japan). The general parameters are presented in Table 4. The sweeps of raw signals, such as IMU data, joint angle, and global positioning of each sensor or anatomical landmark, will be averaged with the time normalized by the percentage of the stride cycle. When the stride cycles cannot be defined because of poor data quality, the dataset will be excluded.

**Table 4:** Items related to the general walking parameters

Spatial parameters	Temporal parameters	Spatiotemporal parameters
Stride length	Cadence	Speed
Base width	Stance-swing ratio	
Step width	Double support time	
Minimum toe clearance		
Joint angles (hip, knee, and ankle)		
Left-right asymmetry		

#### Psychological analysis

A cognitive performance is evaluated by reaction time, process time, and task accuracy. The reaction time will be calculated for the SRT, Go/No-Go task, Stroop Color–Words Test, and N-back test. The reaction time is defined as the interval between the onset of the target signal and the participant’s response. The process time is defined as the interval between the task onset and completion in the TMT and DS test. Furthermore, we will compare the cognitive performance with the scores of the conventional paper-based assessments (MMSE and MoCA-J) using multiple linear regression analysis in order to confirm the validity of our custom-made application.

### Statistical analysis

In this study, we will establish statistical models for predicting cognitive functions based on walking characteristics in healthy participants. At first, we will exclude the data obtained by MCR participants for the statistical analysis. MCR will be defined using the algorithm by Marquez et al. [33]. According to this study, we defined MCR syndrome as slower comfortable walking speed (-1SD below the mean of whole dataset) and subjective memory complaint as detected by the GDS-15. After the exclusion of the data obtained from MCR participants, we will perform the statistical analyses using the following processes.

The dependent variables are reaction time, process time, and task accuracy, and the independent variables are walking parameters and averaged signal traces, in both the fast and comfortable conditions. Prior to the substitution of the independent variables in the statistical model, we will sift the variables, to prevent problems related to multi-covariance [51].

The walking parameters will be reduced to less than 25 dimensions, by principal component analysis (PCA), as needed. The statistical model will be used for multiple linear regression, with and without the random sample consensus (RANSAC) algorithm. The accuracy of the statistical model will be evaluated using Akaike’s information criterion (AIC) [52]. All statistical analyses will be performed using the Python script, with the *scikit-learn* library.

## Discussion

To the best of our knowledge, this will be the first study to use a three-dimensional motion capture system to reveal the relationship between walking and cognitive functions in healthy adults. This database will be essential for developing a system that detects early cognitive risks based on walking performances in a daily environment. As described in the “Introduction” section, the conventional optical motion capture system requires more than three cameras and several reflective markers, which are located in laboratory environments and not daily situations. Thus, the IMU-based motion capture system is more suitable for the objectives of this study as it can be embedded into wearable devices, such as the e-skin MEVA in this study.

From a methodological perspective, the accuracy of the algorithm for three-dimensional bone modeling in the e-skin MEVA has already been confirmed in a previous study and was also supported by the findings of our preliminary experiment, comparing it with conventional motion capture systems (see Supplementary Figure S2, Additional file 1) [36]. In fact, the e-skin MEVA and LETS WALK have already been applied in clinical fields to detect abnormal walking patterns owing to spinal cord injury (Higashibaba and Irie, in submission). Thus, we believe that the accuracy of three-dimensional motion capture, using the e-skin MEVA, is adequate for this study. Furthermore, the raw IMU data during walking will be published with our study database, which would enable users and developers of other IMU devices to use the database for their own healthcare applications. However, it is important to assess the validity of the IMU-based system compared to the conventional optical motion capture system to improve the comparability of the database. Thus, we also plan future validity studies of the e-skin MEVA in multiple locations and to publish those datasets simultaneously as the database from this study.

For cognitive assessments, we used both the custom-made web-based- and paper-based cognitive assessments in this protocol. As described in the “Introduction” section, conventional paper-based testing methods, such as MMSE and MoCA, are not suitable for this study because of their ceiling effects in healthy participants [23–26]. Moreover, web-based cognitive assessment has recently gained attention because of its feasibility in detecting early stage cognitive decline compared to paper-based testing [27].

In particular, our custom-made application could better assess higher dimensional cognitive performances, including the temporal, spatial, and spatio-temporal domains, than conventional applications. Additionally, we will obtain scores of the conventional paper-based assessments (MMSE, MoCA-J, and GDS-15). These scores will be used for the screening of pre-diagnostic dementia, MCI, and MCR and for comparison with variables obtained by the web-based application for validity testing, which will assist further studies aiming to detect MCI and MCR.

The associations between walking and cognitive functions have been well described in studies on cognitive interference in walking. Killeen et al. reported that minimum toe clearance decreased while a Stroop Color-Word test was administered under experimental conditions [53]. In addition, there have been several reports about such interferences, assessed using the well-known dual task paradigm [54–56], considered to be compensatory mechanisms and/or overlapping functional localization [57]. These common neural mechanisms in walking and cognition are related to changes in walking characteristics that precede cognitive decline. However, the relationship between these interferences in dual-task paradigms and natural walking parameters without any cognitive loading is unclear. Further studies are required to elucidate this relationship.

This study has several limitations. First, although we checked the reliability of the e-skin MEVA compared to the conventional motion capture system, there are several errors in the three-dimensional model calculation because the MEVA algorithm calculates a three-dimensional model based on gender and body height, not including the length of each segment [36]. Moreover, the accuracy of the IMU-based motion capture system relies on the pitch (cadence) of walking [58]. Second, our cognitive assessment tools have not been compared to conventional tools, such as the MMSE and MoCA, which might decrease the reliability of the overall experiment from a methodological perspective.

For gait measurement, we posit that this study focuses on revealing the relationship between walking and cognitive functions in healthy participants. We also aim to utilize the database to detect early cognitive risks for MCI and MCR patients from walking in a daily environment, which will be realized by further studies. Thus, the IMU-based motion capture system is more suitable for gait measurement in this study than the conventional optical motion capture system from the perspective of the daily monitoring, even though the accuracy of the IMU-based system is inferior to the conventional optical system [59]. Additionally, it is important that the comparison between the IMU-based (e-skin MEVA) and conventional optical systems is published on open repositories in order to assure compatibility with other measurement systems. In this study, we will publish the dataset of this comparison simultaneous to the publication of the whole database, which will improve the utility of the database so that it will be available for any device and environment [60].

For cognitive assessments, our preliminary experiments indicated a significant relationship between parts of walking and cognitive parameters (see Supplementary Figure S3, Additional file 1) [42, 48, 60, 61]. Additionally, our web-based applications were created based on a well-established psychological paradigm that is sensitive to early cognitive decline, even in healthy participants, in whom functional localizations and relationship between cognitive impairments have already been detected [62–70]. Thus, we believe that our cognitive assessment application has adequate reliability to detect the relationships between the walking and cognitive functions [42, 48, 62, 63]. Additionally, we will compare the results of the paper-based and our custom-made application in this study. Thus, it will allow us to observe the compatibility, superiority, and inferiority of our application comparing with conventional paper-based tests.

For clinical implications, the database developed in this study could provide standard values for both walking and cognitive functions, which would support the evaluation of psychomotor function, including the MCR syndrome. Our research team also plans to conduct a cohort study for participants, to define the risks of MCR and MCI. This CoGait project database will be developed for use as a worldwide platform, for cross-sectional and longitudinal studies on cognition, walking, and frailty, as well as other studies in the field of geriatrics.

## Supplementary Information

**Additional file 1: **Preliminary results. We performed preliminary experiments before developing this study protocol. **Figure S1.** depicts the result of a pilot observation of the relationship between the walking and cognitive parameters in a small population. **Figure S2.** shows the result of comparisons of joint angles between MEVA and VICON (conventional motion capture system). **Figure S3.** presents the results of preliminary experiments of cognitive performance using a custom-made web-based application. **Figure S4.** shows the rule for the Japanese *kana* letters in the TMT-B test.

**Additional file 2.** STROBE checklist. The STROBE checklist for cross-sectional studies. Each cell in the line numbers column indicates the line numbers within the manuscript where each item of the checklist is located.

## Data Availability

After publication, the database of walking and cognitive parameters developed in this study will be published as a public repository. Access permissions will be managed by the Tokyo University of Technology and Xenoma Inc.

## Abbreviations

AIC: Akaike’s information criterion
DS: Digit span
GDS-15: Geriatric Depression Scale 15
IMU: Inertial measurement unit
MCI: Mild cognitive impairment
MCR: Motoric cognitive risk
MMSE: Mini-Mental State Examination
MoCA: Montreal cognitive assessment
RANSAC: Random sample consensus
SRT: Simple reaction time
TMT: Trail Making Test
