# Supplementary material for: The relationship between spatiotemporal gait parameters and cognitive function in healthy adults: protocol for a cross-sectional study
Source: Pilot Feasibility Stud. 2022 Jul 25;8:154. doi: 10.1186/s40814-022-01122-z (PMC9310397; doi:10.1186/s40814-022-01122-z)
Supplement: Supplementary file 1 — Additional file 1: Preliminary results. We performed preliminary experiments before developing this study protocol. Figure S1. depicts the result of a pilot observation of the relationship between the walking and cognitive parameters in a small population. Figure S2. shows the result of comparisons of joint angles between MEVA and VICON (conventional motion capture system). Figure S3. presents the results of preliminary experiments of cognitive performance using a custom-made web-based application. Figure S4. shows the rule for the Japanese kana letters in the TMT-B test. [file 40814_2022_1122_MOESM1_ESM.docx]

Figure S1. Preliminary experiment of the walking and cognitive parameters

The color maps indicate Pearson’s correlation coefficients between each walking and cognitive parameter. The left color map indicates all combinations of the pairs; on the right are the extracted pairs, in which the *p*-values were smaller than the significance level (0.05). The datasets were obtained from healthy volunteers aged 28–80 years. The walking parameters were obtained using the same methods as in this study protocol, but the cognitive functions were measured using other software (CogEvo Pro, Total Brain Care, Kobe, Japan). ASI: asymmetry index.

Figure S2. Comparisons of joint angles between MEVA and VICON

Typical joint angle recordings from both sides of the hip, knee, and ankle were obtained from a single participant, using e-skin MEVA (red traces) and VICON (blue traces) while the participant walked on a straight walkway under comfortable conditions.

Figure S3. Preliminary experiment of cognitive performances using a web-based application

The dataset of preliminary experiments for digital-based cognitive assessments was obtained from four (A, D, and E) and five participants (B and C). (A) Reaction times of SRT, Go/No-Go, and Stroop Color–Word tests obtained from four participants. (B and C) Accuracy (B) and reaction times (C) of the N-back test obtained from five subjects. (D) Process time for TMT-A (red traces) and TMT-B (blue traces). The process time was expressed as the cumulative time from when the first button is pushed. (E): Matching rate of numerical digits in the DS test for forward (red traces) and backward conditions (blue traces).

Figure S4. The rule for the Japanese *kana* letters in the TMT-B test

Japanese *kana* letters are a common phonogram in the Japanese language. The ascending order of *kana* letters is expressed as shown in the figure. In this experiment, we used 12 letters, from あ[a] to し[shi].
